# Transcriptional networks shaping malting quality in barley: From grain development to brewing performance

**DOI:** 10.1016/j.fochms.2025.100348

**Published:** 2025-12-30

**Authors:** Bahman Panahi, Rasmieh Hamid, Zahra Ghorbanzadeh, Saber Golkari, Mehmet Yildirim, Feba Jacob

**Affiliations:** a Department of Genomics, Branch for Northwest & West region, Agricultural Biotechnology Research Institute of Iran (ABRII), Agricultural Research, Education and Extension Organization (AREEO), Tabriz 5156915-598, Iran; b Department of Plant Breeding, Cotton Research Institute of Iran (CRII), Agricultural Research, Education and Extension Organization (AREEO), Gorgan, Iran; c Department of Systems Biology, Agricultural Biotechnology Research Institute of Iran (ABRII) Agricultural Research Education and Extension Organization (AREO), Karaj, Iran; d Field Crops Department, Agricultural Faculty, Dicle University, Diyarbakir, Turkiyeh; e Centre for Plant Biotechnology and Molecular Biology, Kerala Agricultural University, Thrissur, India

**Keywords:** Hormonal signaling networks, Endosperm modification, Β-Glucan degradation, Genome editing targets, Systems-level malting regulation

## Abstract

Barley (*Hordeum vulgare* L.) is a cornerstone of the malting and brewing industry, yet the molecular regulation of its key quality traits remains incompletely understood. While the biochemical mechanisms governing starch metabolism, storage protein turnover, β-glucan remodeling, and hydrolytic enzyme activity are well characterized, the transcriptional networks orchestrating these processes during grain development and germination remain less defined. This review hypothesises that transcription factors (TFs) serve as central regulatory hubs, integrating hormonal signals with metabolic pathways to modulate malting quality. Advances in functional genomics, transcriptomics, and network biology increasingly support this model, highlighting the roles of MYB (e.g., GAMYB), DOF, bZIP, NAC, WRKY, and AP2/ERF TFs in regulating starch biosynthesis, endosperm protein dynamics, cell wall degradation, and enzyme induction, particularly under gibberellin–abscisic acid crosstalk. Multi-omics integration, weighted gene co-expression network analysis, and natural allelic variation have identified key regulatory modules associated with malt extract yield, fermentability, free amino nitrogen, and wort viscosity. These insights offer promising targets for genome editing, predictive breeding, and synthetic modulation of malting pathways. By linking TF biology to critical brewing performance traits, this review presents a mechanistic framework for leveraging these findings to develop climate-resilient barley cultivars with consistent and enhanced malting quality, paving the way for innovations in malting science.

## Highlights

• TF networks regulate starch mobilization and β-glucan breakdown, shaping malting efficiency.
• Regulatory hubs identified as key drivers of malt extract yield and overall fermentability.
• Hormone-responsive TF modules orchestrate germination timing and enzyme induction dynamics
• Genome editing targets revealed to enhance malting quality and brewing performance outcomes.

## 1 Introduction

Barley (*Hordeum vulgare* L.) underpins the global malting and brewing industry owing to its uniquely favorable combination of high starch content, optimal endosperm architecture, and intrinsic hydrolytic enzyme potential. These attributes govern the production of fermentable sugars, proteolytic products, and flavour precursors that together determine brewing performance and finished beer quality (Alvi & Saleem, 2024). Although the biochemical pathways underlying starch degradation, storage-protein mobilization, and β-glucan breakdown are well established, the transcriptional networks that coordinate these processes are far less understood. Increasing evidence indicates that malt quality emerges not from enzyme abundance alone, but from the finely tuned transcriptional regulation of genes controlling reserve mobilization, hormone signaling, and cell-wall remodeling (Tian et al., 2025). A limited understanding of these regulatory mechanisms restricts our ability to predict and optimize malting performance under genetic and environmental variation.

Malting is a controlled germination process comprising soaking, germination, and kilning, each associated with distinct physiological and molecular transitions (Fox & Bettenhausen, 2023). During soaking, kernels typically reach 42–48 % (*w*/w) moisture, a range consistent with both commercial and micromalting conditions that is sufficient to trigger metabolic reactivation and gibberellic acid (GA)-mediated signaling cascades (Almaguer et al., 2024; Fox & Bettenhausen, 2023; O'Lone et al., 2024). GA perception through Gibberellin Insensitive Dwarf1 (GID1) receptors promotes DELLA degradation, enabling the activation of transcription factors such as GA -Induced MYB (GAMYB) that induce genes encoding hydrolytic enzymes essential for endosperm mobilization (Shewry & Darlington, 2024). Concurrently, β-glucan degradation begins to weaken endosperm cell walls and permit the movement of enzymes and substrates (Fan et al., 2025). Germination is characterized by a strongly coordinated rise in α-amylase, β-amylase, limit dextrinase, β-glucanases, and proteases, which collectively generate fermentable sugars and free amino nitrogen (FAN) (Li, Cai, Zheng, Zhang, and Ye, 2023). Transcriptomic analyses show upregulation of more than 25 starch-degrading genes including *Amy1* and *Amy2* isoforms together with proteases whose expression is synchronized with storage-protein breakdown, underscoring the central role of transcriptional control in malt modification (Collins et al., 2021, Kumar, Chaturvedi, and Singh, 2023, Zhao et al., 2023).

**Fig. 1:**
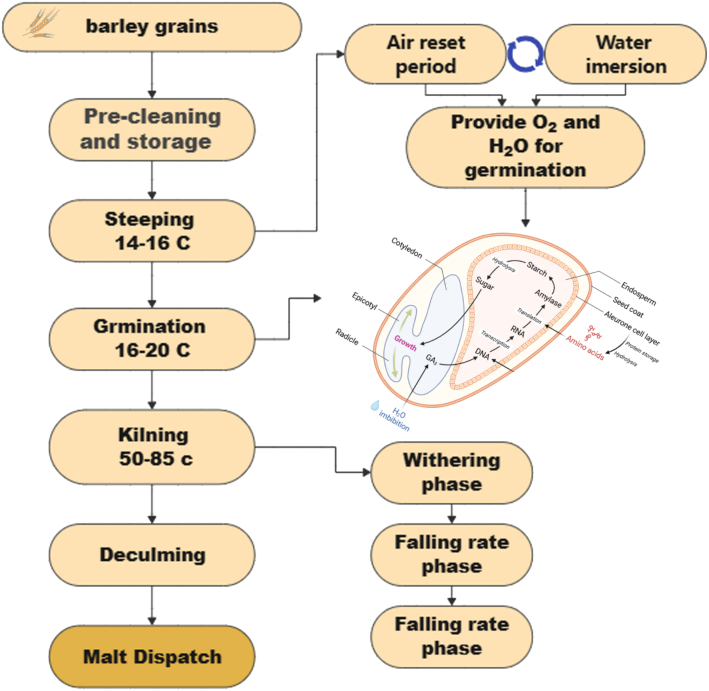
Overview of the malting process and transcriptional regulation during grain germination. The schematic illustrates the major stages of the industrial malting process, beginning with barley grain pre-cleaning and storage, followed by steeping (alternating water immersion and air rests to supply O₂ and H₂O), germination (16–20 °C) where hormonal signaling and transcriptional reprogramming occur, and kilning (50–85 °C) to arrest germination while preserving hydrolytic enzymes. The subsequent deculling and malt dispatch steps prepare the final product for brewing. The inset depicts the transcriptional and biochemical regulation within the germinating grain: gibberellic acid (GA) produced in the embryo triggers the aleurone layer to activate transcription factors (e.g., GAMYB), which in turn induce genes encoding hydrolases such as α-amylases, proteases, and β-glucanases. These enzymes mobilize starch and storage proteins into fermentable sugars and amino acids, generating free amino nitrogen.

Kilning, the final stage, arrests germination through controlled dehydration while shaping enzyme stability and imparting characteristic sensory attributes to the malt (Shewry & Darlington, 2024). The thermal regime affects enzyme groups differentially: during early drying (∼45–55 °C), gradual moisture reduction allows partial retention of existing enzymes like α-amylase, β-amylase, limit dextrinase, and specific proteases, while certain enzymes, including hydrolases, are newly synthesized or activated as metabolism declines(Marčiulionytė et al., 2022). As temperatures rise to 80–85 °C, Maillard reactions generate melanoidins responsible for colour and flavour (Rani & Bhardwaj, 2021),), while continued heating gradually inactivates most enzymes. In contrast, thermostable β-amylase isoforms and a subset of proteases are retained, maintaining their activity. Importantly, these kinetics apply predominantly to pale malt production: in the production of darker and specialty malts, considerably higher curing temperatures (100–220 °C) are applied, which severely diminish enzymatic potential despite enhancing sensory complexity (Fox & Bettenhausen, 2023). The higher temperatures in darker malts also result in more pronounced colour and flavour development but further reduction in enzymatic activity, which affects the potential for fermentation and mashing. Thus, the final enzymatic composition of malt reflects the balance between thermal protection during early drying and progressive heat-induced denaturation during curing, with a strong dependence on malt type and temperature regime (Fig. 1).

Comparative studies demonstrate that enzymatic activities underpin key malting traits: α-amylase strongly correlates with extract yield, FAN, wort viscosity, and the Kolbach Index (KI), while β-amylase is a major determinant of diastatic power. β-glucanase improves wort filtration and protein solubility, illustrating the synergistic action of hydrolases during modification (Cai et al., 2025). These traits correspond to differential expression of *HvAmy*, *HvBmy*, and *HvGlb* gene families and are modulated by kernel characteristics such as grain size uniformity, starch granule architecture, and protein content (Fox & Bettenhausen, 2023). Starch granule architecture plays a key role in malting performance because the different types of starch granules A-type and B-typehave distinct physical properties. The relative proportions of these granules determine how easily the starch can be gelatinised and accessed by enzymes during germination. Specifically, B-type granules, which have a higher surface area and lower crystallinity, are more readily hydrolysed during germination, thus contributing disproportionately to fermentable sugar release (Sun et al., 2022). Similarly, protein content influences malting performance by affecting hydration and the breakdown of storage proteins. High protein content, especially the hordein-rich protein matrix surrounding starch granules, can impede water uptake and enzyme diffusion, slowing the malting process. In contrast, moderate protein levels facilitate protease activity, which supports efficient breakdown of storage proteins and promotes the production of FAN during malting (Gebre et al., 2025; Jaeger et al., 2021).

Despite considerable biochemical knowledge, the transcriptional architecture governing malting quality remains incompletely resolved. Recent transcriptomic studies highlight that transcription factors orchestrate hydrolase induction within defined developmental windows (Vinje et al., 2021), and multi-omics analyses under abiotic stress demonstrate that environmental perturbation disrupts regulatory networks associated with malt quality (Hong et al., 2020). Weighted Gene Co-expression Network Analysis (WGCNA) has begun to identify transcription factors regulating key malting-related genes, providing preliminary insights into hierarchical gene-regulatory modules influencing quality traits (Li, Cai, Zheng, Zhang, and Ye, 2023). Thus, the current challenge lies in mapping these gene regulatory networks to improve malting performance through breeding and functional genomics. This review aims to resolve the transcriptional networks and key transcription factors responsible for starch degradation, storage-protein mobilization, and β-glucan remodeling, integrating functional genomics and co-expression network analysis to provide mechanistic insights for the development of barley cultivars with improved malting performance and resilience.

## 2 Molecular basis of grain quality traits

Grain quality in malting barley is primarily dictated by the quantity and structural properties of starch, the composition and solubility of storage proteins, the concentration and fine structure of (1,3;1,4)-β-glucans, and the intrinsic capacity for hydrolytic enzyme synthesis. These molecular features directly determine extract yield, fermentability, and wort viscosity/filtration performance during brewing. For example, elevated β-glucan content increases wort viscosity and impairs separation efficiency and therefore must be evaluated in a trait-integrated framework (Fox & Bettenhausen, 2023; Langenaeken et al., 2020; Rani & Bhardwaj, 2021).

These biochemical traits are under tight genetic (G) regulation but are strongly modulated by environmental (E) factors, with the combined effect of genotype-by-environment interaction (G × E) further influencing their expression. Multi-environment trials reveal significant contributions of genotype, environment, and G × E interactions to malting quality, while time-resolved transcriptomic analyses during micromalting demonstrate synchronized activation of starch- and cell wall–degrading enzymes and their transcriptional regulators. Such findings justify a detailed focus on transcriptional and network-level regulation of starch metabolism, storage-protein turnover, β-glucan remodeling, and hydrolase capacity in the sections that follow (Cai et al., 2025; Ramanan et al., 2023; Vinje et al., 2021).

## 3 Starch biosynthesis and modification

**Fig. 2:**
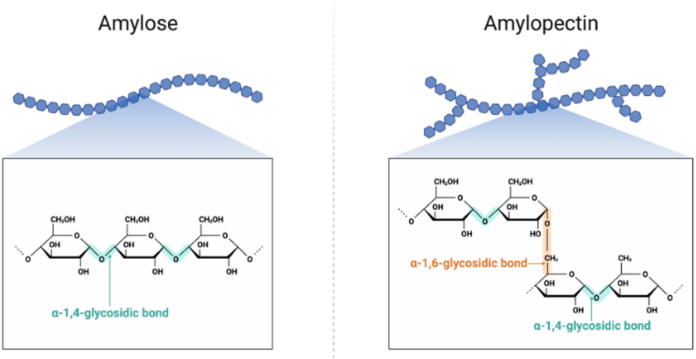
The schematic illustrates Structure of starch.

Starch constitutes over 60 % of the dry weight of barley grain and serves as the principal fermentable reserve for brewing. Structurally, it comprises two polysaccharides: amylose, a predominantly linear α-1,4-glucan, and amylopectin, a highly branched α-1,4/α-1,6-glucan (Fig. 2) (Gielens et al., 2024). The amylose-to-amylopectin ratio and granule-size distribution are critical determinants of starch functionality, influencing gelatinisation behaviour, enzymatic hydrolysis, and ultimately malt-extract yield and fermentability (Chang et al., 2025; Varghese et al., 2022).

Barley starch is also heterogeneous in granule morphology, consisting of large lenticular A-type granules and small spherical B-type granules. A-type granules dominate starch mass but are relatively less accessible to enzymatic hydrolysis, whereas B-type granules are more readily degraded during malting, contributing disproportionately to fermentable sugar release (Li, Ahmad, et al., 2024). The relative abundance of A- and B-type granules is therefore a critical determinant of malt modification and brewing efficiency. The biosynthesis of starch in the barley endosperm is orchestrated by a coordinated network of enzymes encoded by multigene families. The committed step is catalyzed by adenosine diphosphate (ADP)-glucose pyrophosphorylase (AGPase), notably the large subunit *HvAGP-L1*, which generates ADP-glucose, the activated glucosyl donor for starch assembly (Huang, Tan, Zhang, Li, and Liu, 2021, Panahi & Jalaly, 2025). Subsequent chain elongation is mediated by granule-bound starch synthase I (GBSSI), which specifically synthesises amylose within starch granules. Allelic variants of *HvGBSSI* significantly alter amylose content and starch structure, with direct implications for malting quality (Farooq, Ma, Shen, and Gu, 2022).

**Fig. 3:**
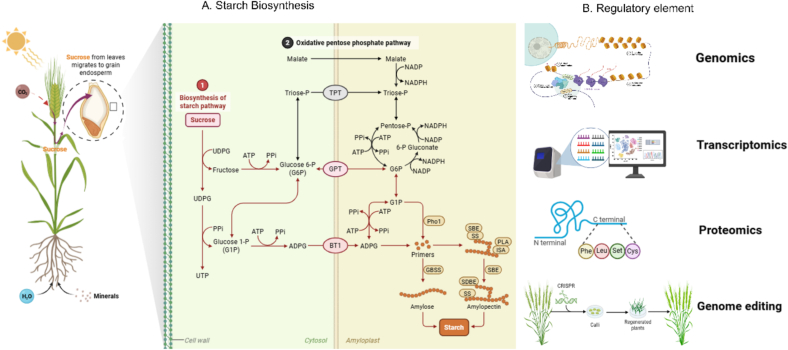
Integrated regulation of starch biosynthesis and multi-omics approaches for improving barley malting quality. (A) Starch biosynthesis in barley endosperm. Photosynthetically derived sucrose from leaves is translocated to the developing grain, where it enters the cytosolic sucrose-to-starch pathway. 1, sucrose synthase; 2, UDP-glucose pyrophosphorylase; 3, AGPase; 6, complexes including AGPase, starch synthase IIa (SSIIa), SSIII, starch branching enzyme IIb (SBEIIb), and SBEIIa; 7, glucose-6-phosphate dehydrogenase. (B) Regulatory elements and omics-driven dissection of starch metabolism. Genomics and transcriptomics identify regulatory genes and expression networks controlling carbohydrate metabolism, while proteomics resolves enzyme abundance, isoform specificity, and post-translational modifications. Genome editing tools such as CRISPR/Cas enable targeted manipulation of key regulators and biosynthetic enzymes, providing opportunities to fine-tune starch architecture. Collectively, these approaches link transcriptional regulation with starch quality traits, offering a systems-level framework for developing barley varieties with improved malting and brewing performance.

Amylopectin synthesis and branching patterns are regulated by starch branching enzymes (SBE), including *HvSBEIIa* and *HvSBEIIb*, and by debranching enzymes, which modulate the frequency and distribution of branch points. These activities define the fine structure of amylopectin, thereby shaping granule crystallinity, gelatinisation temperature, and enzymatic accessibility during malting (Baysal et al., 2020).). Natural allelic diversity at *HvSBEIIa* has been shown to modify granule morphology, influencing extract yield and fermentation efficiency (Fig. 3A) (ElShamey et al., 2025).

Advances in functional genomics and genome editing have clarified the molecular determinants of starch biosynthesis (Fig. 3B). For example, clustered regularly interspaced short palindromic repeats/CRISPR-associated protein 9 (CRISPR/Cas9)-mediated knockout of HvGBSSI reduces amylose content, producing starch with altered gelatinisation and enhanced modification efficiency without yield penalties (Wang, Hsu, Wu, Yeh, and Ku, 2021, Zhou, Ding, et al., 2025). Similarly, knockout of the ethylene-responsive factor (ERF) gene *HvERF72* enhances B-type granule formation and increases total starch content; transcriptional profiling at 15 days after flowering (15 DAF) revealed coordinated upregulation of *HvAGPL1*, *HvAGPS1*, *HvSS2a*, *HvSBEI*, *HvSBEIIb*, and *HvGBSSI* in the mutants s (Zhou, Ding, et al., 2025). These findings demonstrate the potential of targeted interventions to fine-tune starch composition and granule architecture for improved malting performance.

Beyond enzymatic pathways, transcriptional regulation plays a central role in modulating starch biosynthesis. Transcriptomic studies in cereals have identified DNA-binding with one finger (DOF) and basic leucine zipper (bZIP) transcription factors that activate genes encoding AGPase and starch synthases, MYB regulators that influence amylose–amylopectin ratios, and NAM/ATAF/CUC (NAC) transcription factors that integrate environmental stress cues with carbohydrate metabolism (Li, Cai, Zheng, Zhang, and Ye, 2023, Li, Tan, and Zhang, 2021). These transcriptional regulators form interconnected networks that align starch deposition with grain development and environmental signals, underscoring their importance as breeding targets for malting quality (Cao et al., 2024). Transcriptomic analyses across barley developmental stages reveal dynamic regulation of starch biosynthetic genes in response to developmental and environmental signals, providing insight into the gene networks controlling starch accumulation and its integration with protein and cell-wall metabolism (Bian et al., 2019). Because starch and protein deposition are closely coordinated during grain filling, the regulation of storage protein composition represents the next critical dimension of malting quality, as discussed in the following section.

## 4 Protein composition and nitrogen utilization

Barley storage proteins, predominantly hordeins, are a major nitrogen reservoir that strongly determines malting and brewing quality (Shewry & Darlington, 2024). These proteins constitute approximately 8–12 % of grain dry weight and accumulate mainly during seed maturation. Hordeins are classified into B-, C-, D-, and γ-type hordeins, encoded by large multigene families dispersed across the barley genome. Their enrichment in proline and glutamine residues underlies both structural properties and functional roles in endosperm development and malting (Fox & Bettenhausen, 2023).

At the molecular level, hordein biosynthesis is controlled by a multi-layered transcriptional network operating with strict spatial and temporal specificity. DOF and PBF (prolamin-box binding factor) proteins are central regulators (Huo et al., 2025). They recognize conserved cis-elements such as the prolamin box and endosperm box in hordein promoters, thereby coordinating transcriptional activation during endosperm development. In barley endosperm, the DOF factor barley prolamin-box binding factor (BPBF) binds directly to the prolamin box (5′-TGTAAAG-3′) of Hor2 promoters, while bZIP transcription factors (BLZ1/BLZ2, O2-like homologs) interact with general control nonderepressible 4 (GCN4)-like motifs, often synergising with BPBF. GAMYB, a gibberellin-induced MYB transcription factor, further enhances this cascade, establishing a barley-specific module for hordein gene activation (Ruta et al., 2020). MYB regulators also contribute by influencing promoter activity and chromatin accessibility, underscoring the complexity of storage protein transcriptional control (Vinje et al., 2021, Zhao et al., 2023). This network ensures the correct formation of protein bodies in the endosperm, which act as nitrogen and amino acid reservoirs essential for seedling growth and malting.

Post-translational modifications (PTMs), particularly phosphorylation, add functional diversity by altering hordein solubility and protease accessibility. Recent proteogenomic studies demonstrated that specific isoforms and PTMs affect degradation kinetics during malting, thereby modulating FAN release (Bobalova et al., 2023). FAN is a central brewing metric, serving as the primary nitrogen source for yeast and a key determinant of fermentation efficiency.

Proteolytic degradation of storage proteins is mediated mainly by cysteine proteases and carboxypeptidases. Their expression is developmentally programmed and induced by germination signals. For example, *Hordeum vulgare* papain-like cysteine protease 1 (HvPap-1) and *HvPap-16* are strongly upregulated during early germination, correlating with enhanced protein solubilisation (Diaz-Mendoza et al., 2019). This programme is hormonally regulated: embryo-derived GA induce GAMYB-dependent activation of protease genes in the aleurone, whereas abscisic acid (ABA) antagonises this pathway, thereby tuning FAN release (Collin et al., 2021; Liang et al., 2025). Proteolysis generates soluble peptides and amino acids, increasing FAN and supporting yeast growth during fermentation.

Brewing-relevant nitrogen metrics include FAN and the Kolbach Index (S:T, ratio of soluble to total protein). Industry practice typically targets ≥150 mg L^−1^ FAN in standard worts and uses S:T to assess the extent of protein modification (Rani & Bhardwaj, 2021). Hordein isoform composition and PTMs influence protease accessibility, thereby shifting FAN and S:T without necessarily altering total protein content.

Genetic variation in transcription factors regulating hordein synthesis and protease activity represents an important breeding lever. Mutations in the barley PBF (BPBF) gene, such as the *lys3.a* allele, causes reduced hordein accumulation and also downregulates β-amylase (*Bmy1*) during grain development, linking PBF functionality to both storage-protein deposition and nitrogen mobilization pathways (Bahmani et al., 2023; Vinje & Simmons, 2024). At the same time, natural variation at *Bmy1* modifies enzyme activity and thermostability, shaping diastatic power and indirectly influencing the nitrogen–carbohydrate balance during mashing (Hornink, 2024; Iqbal, 2022). Biotechnological approaches targeting these regulators or protease genes may allow optimization of both proteolysis and consistency in malt quality. Historically, malting and brewing industries selected barley primarily by total protein content (9–13 %) due to its inverse relationship with starch accumulation. Lower protein typically correlates with higher starch, favoring fermentable sugar yield (Langenaeken et al., 2020). However, environmental stress can disrupt this balance by altering transcriptional programs of both starch and protein metabolism, thereby reducing starch deposition and impairing malt quality (Kim & Kim, 2021, Li, Cai, Zheng, Zhang, and Ye, 2023).

Although total protein remains a key quality indicator, it obscures the molecular complexity of the barley proteome. Modern proteomics confirms that hordeins and high-molecular-weight glutelins, alcohol- and reducing agent–soluble proteins rich in proline and glutamine, dominate the endosperm storage fraction (Fox & Bettenhausen, 2023). They account for 40–70 % of total protein, with proportions shaped by genotype and environment (Bose et al., 2021). Despite their clear impact on hydration kinetics and malt modification, endosperm storage protein composition is not yet widely adopted as a breeding trait. Additional proteins, such as hordoindolines, affect grain hardness by modulating protein–starch interactions; in barley, their effects are allele-dependent and generally weaker than wheat puroindolines, but null alleles (e.g., HinB-2) can increase grain hardness and alter hydration and modification kinetics (Dinsa, 2023; Kalra et al., 2019). Moreover, β-amylase, a starch-degrading enzyme expressed during grain filling and persisting into germination, exhibits co-regulation with storage protein genes, linking starch degradation capacity with nitrogen metabolism and diastatic power (Apriyanto & Fettke, 2025).

## 5 β-glucan accumulation and degradation

β-glucans are key determinants of malting and brewing quality, as their concentration and structure directly influence wort viscosity, filtration efficiency, and extract yield (Blšáková et al., 2021). These (1,3;1,4)-β-glucans, also referred to as mixed-linkage glucans, are the principal non-starch polysaccharides in the cell walls of barley endosperm and aleurone tissues (Held, 2022). A detailed molecular understanding of their biosynthesis and degradation is therefore essential for developing barley cultivars with superior malting performance. Biosynthesis of (1,3;1,4)-β-glucan is controlled by the cellulose synthase-like (Csl) gene families *HvCslF* and *HvCslH*. Among these, HvCslF6 is firmly established as the primary determinant of β-glucan accumulation in developing grains (Gimenez, 2019). Garcia-Gimenez et al. (2020) employed CRISPR/Cas9 to generate knockouts of *HvCslF6*, *HvCslF3*, *HvCslF9*, and *HvCslH1*. *HvCslF6* mutants completely lacked (1,3;1,4)-β-glucan in mature grain, confirming its non-redundant role. In contrast, *HvCslF3* and *HvCslH1* knockouts showed no measurable impact on β-glucan content, suggesting minor or stage-specific functions. *HvCslF9* mutants retained wild-type β-glucan levels but exhibited altered profiles of other wall polysaccharides, highlighting more subtle contributions to cell wall architecture. Loss of *HvCslF6* also caused morphological alterations, including reduced thousand-grain weight, modified grain dimensions, and decreased surface area, underscoring the trade-offs associated with manipulating this locus (Garcia-Gimenez et al., 2020). Importantly, natural allelic variation at *HvCslF6* and its regulatory elements represents a valuable molecular resource for breeding barley lines with optimized β-glucan levels tailored for brewing applications (Lante et al., 2023).

Degradation of β-glucan during malting is equally critical. Endo-(1,3;1,4)-β-glucanases, induced during germination via GA signaling, hydrolyze endosperm walls to reduce wort viscosity and improve filtration (Edison et al., 2022). Precise temporal activation of glucanase genes ensures controlled cell wall degradation during steeping and germination. Transcriptomic and proteomic studies have shown that glucanase expression is tightly coordinated with physiological changes in the grain, emphasizing integrated regulation of β-glucan breakdown (Geng et al., 2022).

## 6 Hydrolytic enzyme potential

A hallmark of malting barley is its capacity to generate a tightly coordinated suite of hydrolytic enzymes most notably α-amylase, β-amylase, limit dextrinase, proteases, and β-glucanases that collectively convert complex storage macromolecules into fermentable sugars and assimilable nitrogen (Kumar, Narwal, Verma, and Singh, 2022). The timing, efficiency of expression, and thermostability of these enzymes critically influence malting quality, fermentation dynamics, and brewing performance.

α-Amylase, encoded mainly by the Amy1 and Amy2 loci, is an *endo*-acting enzyme that catalyzes the cleavage of internal α-1,4-glycosidic bonds in starch molecules, thereby initiating hydrolysis after gelatinization. In contrast, β-amylase (Bmy1) functions as an *exo*-enzyme, sequentially releasing maltose from the non-reducing ends of starch polymers (Nag et al., 2021; Panahi et al., 2024). Limit dextrinase specifically hydrolyzes α-1,6 branch points in partially degraded amylopectin, enabling complete starch mobilization. Concurrently, cysteine proteases such as HvPap-1 and HvPap-16 degrade storage proteins, particularly hordeins, thereby releasing FAN, a critical nutrient for yeast during fermentation (Geissinger et al., 2022).

The temporal and spatial regulation of these enzymes during steeping and early germination is mediated by phytohormonal signaling networks, particularly GA and ABA **(**Jhanji et al., 2024**)**. GA, synthesized in the embryo following imbibition, binds to the gibberellin-insensitive dwarf 1 (GID1) receptor and promotes ubiquitin-mediated degradation of DELLA repressors. This process activates GA-responsive transcription factors, most notably GAMYB, which binds to GA-responsive elements (GAREs) and amy-box motifs in promoters of α-amylase and other hydrolase genes, thereby driving their transcriptional induction (Ma et al., 2024).

Recent studies have identified cis-regulatory polymorphisms in the promoters of Amy1 and Bmy1 that correlate with increased enzyme activity, thermostability, and malt extract yield (Iqbal, 2022, Wu et al., 2022). These findings reveal the genetic basis of enzymatic efficiency and provide valuable molecular markers for marker-assisted selection (MAS) in barley breeding programs. The thermostability of hydrolytic enzymes has become increasingly important for modern high-temperature mashing protocols. Conventional β-amylase isoforms are thermolabile, rapidly losing activity between 60 and 70 °C, which limits fermentable sugar release during late mashing stages (De Schepper et al., 2022). However, natural variation in barley landraces and wild relatives has yielded thermostable isoforms of both α- and β-amylase. Introgression of these alleles into elite cultivars has produced lines with enhanced enzyme resilience and sustained activity under industrial malting conditions (Rani et al., 2024).

## 7 Transcription factors regulating grain development and quality

### 7.1 Transcription factors (TFs) involved in starch biosynthesis and carbohydrate metabolism

**Fig. 4:**
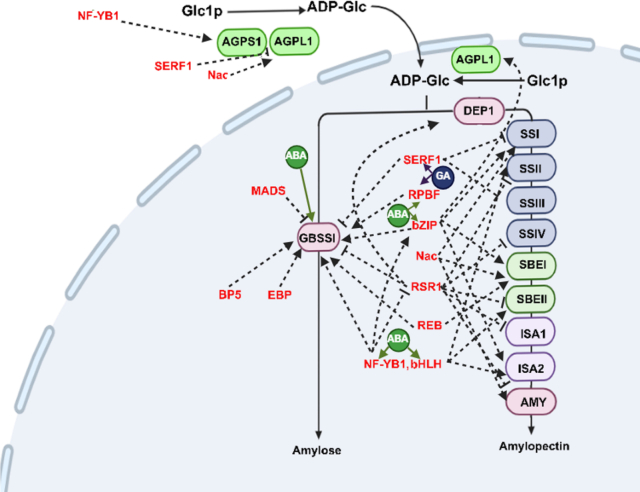
Several key transcription factors (red font) and methylation are involved in the stimulation or inhibition of starch synthesis-related genes. Gibberellin (GA) modulates the expression of SERF1 and RPBF in starch synthesis to promote starch accumulation in the developing barley endosperm. Abscisic acid (ABA) synthesized in barley leaves directly activates the expression of most SSRGs and multiple hub transcription factors. The arrowed lines indicate activation, and the bar-ended lines indicate inhibition. AGPS (ADPG pyrophosphorylase AGPase), AGPL1 (ADPG transporter), GBSSI (Granule-bound starch synthase), SS (starch synthase), SBE (Starch branching enzyme). (For interpretation of the references to colour in this figure legend, the reader is referred to the web version of this article.)

Starch biosynthesis and carbohydrate metabolism are essential processes in cereal grains, directly determining grain yield, quality, and malting performance (Fox & Bettenhausen, 2023, Huang, Tan, Zhang, Li, and Liu, 2021). These pathways are subject to multi-layered transcriptional regulation, with transcription factors (TFs) acting as key controllers of gene expression in response to developmental, hormonal, and environmental signals (Panahi, 2025; Panahi et al., 2024; Song et al., 2024). By binding to specific cis-regulatory elements (cis-elements) in promoters of starch synthesis and carbohydrate metabolism genes, TFs align carbohydrate storage and mobilization with the physiological needs of the developing grain (Fig. 4) (Ding et al., 2021, Li, Tan, and Zhang, 2021).

Among these regulators, the bZIP family has been most extensively characterized. In rice, *OsbZIP58* directly activates Wx (granule-bound starch synthase, GBSS), SBE1 (starch branching enzyme), and AGPL3 (adenosine diphosphate-glucose pyrophosphorylase large subunit)**,** thereby promoting starch granule formation in the endosperm (Cao et al., 2022). These TFs are modulated by sugar levels and phytohormones such as ABA and GA, ensuring coordinated starch deposition. Barley homologs specifically BLZ1 and BLZ2 interact with endosperm-specific motifs including the GCN4-like motif and prolamin box, in storage protein gene promoters and form homo- and heterodimers, indicating conserved regulatory modules in seed gene expression (Contreras et al., 2025).

The NAC family also plays an essential role by linking developmental signals and abiotic stresses with carbohydrate metabolism. Transcriptomic analyses in barley indicate co-expression of NAC TFs with starch biosynthetic genes during grain filling, suggesting that they modulate the balance between starch accumulation and mobilization under variable environmental conditions (Fuertes-Aguilar & Matilla, 2024, Wang and Han, 2025). Additionally, in rice, OsNAC25, OsNAC20, and OsNAC26 form a regulatory loop that fine-tunes starch synthesis by balancing promoting and inhibitory interactions (Wang, Chen, Zhang, Meng, and Wei, 2020, Xu et al., 2024).

R2R3-MYB transcription factors influence starch structure, particularly the amylose-to-amylopectin ratio, which critically affects starch functionality and brewing quality. In maize, MYB proteins regulate branching enzyme and synthase genes, altering granule morphology and digestibility (Zhang, Dong, Ji, Wu, and Messing, 2019). Comparative evidence supports similar functions for barley MYBs, highlighting their potential as breeding targets to optimize malt extract yield and fermentability.

The DOF (DNA-binding with one finger) family comprises seed-preferential regulators that fine-tune carbohydrate metabolism. In maize, ZmDOF36 activates AGP-ase and starch synthase genes, boosting starch deposition (Wu et al., 2019). DOF factors often act cooperatively with bZIPs and other TFs, suggesting a conserved regulatory module across cereals. Barley DOF TFs binding the prolamin box likely partner with bZIPs to regulate endosperm development and starch-protein deposition (Zou & Sun, 2023).

The WRKY family, primarily associated with stress responses, also contributes to starch turnover. Several WRKYs are activated by GA during germination and directly upregulate α-amylase genes, enabling rapid starch hydrolysis for seedling growth (Nie et al., 2022). In barley, WRKY TFs such as SUSIBA2 bind sugar-responsive elements and mediate uniform starch mobilization during malting a critical trait for consistent malt quality (Shao et al., 2025, Zhou, Wang, et al., 2025). ABA-responsive TFs, including the ABA-insensitive (ABI) and ABA-responsive element-binding (AREB) families, likewise modulate carbohydrate metabolism under stress or dormancy (Panahi & Shahi, 2024). These regulators control starch degradation and sugar transport genes, thereby integrating stress responses with carbon allocation to safeguard germination and early seedling vigor under suboptimal conditions (Ayub et al., 2025).

### 7.2 Regulation of storage protein gene expression

Storage proteins in cereal grains such as barley, wheat, and maize play essential roles in seed development, serving as nutrient reserves and significantly influencing quality traits in both food and malting industries (Khalid et al., 2023). These proteins, primarily prolamins (e.g., hordeins in barley) and glutelins, accumulate during seed maturation and function as the principal nitrogen reservoir during germination (Shewry & Darlington, 2024). Their expression is tightly regulated at multiple levels to ensure precise spatial and temporal deposition (Li et al., 2025). At the transcriptional level, storage protein gene expression is controlled by interconnected networks of TFs that integrate developmental programs, hormonal cues, and environmental conditions. The major TF families involved include bZIP, DOF, MYB, and NAC, each binding to distinct cis-regulatory elements within storage protein promoters (Dhatterwal et al., 2024; Fuertes-Aguilar & Matilla, 2024; Razzaq & Du, 2024).

The bZIP family, notably the Opaque-2 (O2) homologs in maize and related cereals, plays a central role in prolamin activation (Chen et al., 2023). These TFs recognize GCN4-like motifs in promoter regions and often cooperate with other TFs to assemble enhanceosome complexes that amplify transcription (Guo et al., 2024). In barley, the seed-specific factors BLZ1 and BLZ2 bind analogous motifs in hordein promoters, driving coordinated protein accumulation during late endosperm development (Contreras et al., 2025; Guo et al., 2024).

The DOF (DNA-binding with one finger) family provides another layer of regulation. DOF TFs bind the prolamin box (P-box) motif in storage protein promoters and often act synergistically with bZIP proteins (Wu et al., 2024). This cooperative interaction fine-tunes expression strength and timing, enabling plasticity in hordein synthesis under developmental and stress-related contexts (Elakhdar et al., 2022; Merlino et al., 2023). MYB transcription factors contribute to storage protein regulation by influencing promoter activity and chromatin accessibility (Wu et al., 2024). Isoform-specific roles have been reported, with certain MYBs functioning as activators and others as repressors, thereby maintaining the balance of storage protein composition (Ma and Constabel, 2019, Wang, Niu, and Zheng, 2021).

NAC transcription factors, classically associated with abiotic stress adaptation, are also integrated into the storage protein regulatory network (Diao et al., 2020). Barley NAC genes show co-expression with hordein biosynthetic genes, suggesting a role in linking nutrient storage with stress-responsive signaling (Xiong et al., 2025). Beyond transcriptional control, post-transcriptional and epigenetic layers add complexity**.** Regulatory mechanisms include mRNA stability, alternative splicing, and microRNAs (miRNAs) (Pandey et al., 2022). For example, nitrogen-responsive miRNAs such as miR827 and miR399 indirectly influence storage protein accumulation by modulating nitrogen metabolism pathways (Das & Sathee, 2023; Paluch-Lubawa et al., 2024). Epigenetic marks, including DNA methylation and histone modifications, further modulate chromatin accessibility and transcriptional activity of hordein genes, supporting stage-specific activation and repression (Che et al., 2025). Hormonal regulation is central to storage protein dynamics. Elevated ABA levels during seed maturation promote prolamin gene activation via ABA-responsive elements (ABREs), while GA at germination repress storage protein synthesis and induce protease expression to mobilize reserves (Kumar, Chaturvedi, and Singh, 2023).

### 7.3 TFs linked to β-glucan and cell wall metabolism

The regulation of β-glucan metabolism and cell wall remodeling during malting in *Hordeum vulgare* involves a highly coordinated transcriptional network (Hong et al., 2020). A recent RNA-seq investigation comparing barley genotypes differing in grain β-glucan content identified differentially expressed genes (DEGs) associated with carbohydrate metabolism, glucan metabolic processes, and hydrolase activity, highlighting molecular drivers of β-glucan synthesis and accumulation during grain development (Garcia-Gimenez et al., 2019; Garcia-Gimenez et al., 2020; Geng et al., 2022). Within this context, TFs modulate the expression of both biosynthetic enzymes, such as HvCslF6 and HvCslH1, and degradative enzymes, including β-glucanases, expansins, and xyloglucan hydrolases (Iqbal, 2022).

An integrative study of malting-induced phenotypic shifts employed weighted gene co-expression network analysis (WGCNA) and protein–protein interaction mapping to identify TFs associated with malt quality traits (Luan, Gao, Wu, et al., 2025). Among these, regulators linked to GA signaling were prominent; GA levels correlated negatively with β-glucan content and positively with the activity of hydrolytic enzymes such as α- and β-amylases (Li, Cai, Zheng, Zhang, and Ye, 2023). This supports a hormonal axis in which GA-responsive TFs indirectly influence cell wall degradation by activating hydrolytic gene networks.

Members of the NAC transcription factor family, including barley homologs of NAC005 and NAC034, are transcriptionally upregulated during early germination, coinciding with the induction of β-glucanases such as Glb1 and Egl1 (Arif et al., 2025; Xin et al., 2025). Their activity likely promotes cell wall loosening and reserve mobilization. MYB TFs, particularly those orthologous to AtMYB61 and AtMYB83, are implicated in regulating cellulose synthase-like genes central to β-glucan biosynthesis (notably HvCslF6) (Garcia-Gimenez et al., 2022). This intersection of biosynthesis and remodeling underlies the dynamic changes in cell wall composition during malting. A summary of the major TF families, their representative genes, targets, and brewing relevance is provided in Table 1.

**Table 1:** Transcription factors regulating β-glucan metabolism and cell-wall remodeling in barley during malting.

TF Family	Representative Genes	Primary Targets / Processes	Function During Malting	Brewing Relevance	References
NAC	HvNAC005, HvNAC034	Glb1, Egl1 (β-glucanases)	Promotes cell-wall loosening and reserve mobilization during early germination	Enhances modification; improves wort filtration	(Li, Cai, Zheng, Zhang, and Ye, 2023)
MYB	HvMYB61 (orthologous to AtMYB61), HvMYB83	HvCslF6 (β-glucan biosynthesis)	Balances β-glucan synthesis and remodeling	Controls β-glucan content; affects wort viscosity	(Li, Cai, Zheng, Zhang, and Ye, 2023); (Zhang, Dong, Ji, Wu, and Messing, 2019)
WRKY	HvWRKY38	β-glucanase genes	Regulates endosperm degradation and polysaccharide breakdown	Reduces β-glucan carry-over into wort	(Zhou, Wang, et al., 2025)
bZIP	ABI5-like proteins	Cell-wall-modifying genes; ABA/GA signaling	Integrates ABA and GA signals to fine-tune hydrolase expression	Coordinates dormancy vs germination; affects enzyme induction	(Collin et al., 2020)
AP2/ERF	Barley ERFs	Hydrolase genes (expansins, glucanases)	Respond to developmental and environmental cues; regulate cell-wall hydrolases	Enhances reserve mobilization; increases malt extract yield	(Collin et al., 2025); (Collin et al., 2025), (Hong et al., 2020)
GA-responsive module	HvGAMYB (indirect regulator)	Hydrolytic enzyme network (α- and β-amylases, β-glucanases)	GA-induced TF cascade coordinating enzyme activation	Improves starch and cell-wall degradation; increases malt extract	(Gimenez, 2019)
Genetic variation	HvCslF6 alleles	β-glucan biosynthesis	Determines baseline β-glucan accumulation	Breeding target for optimized wort viscosity	(Garcia-Gimenez et al., 2020); (Lante et al., 2023)
Transcriptomic validation	WGCNA modules	Co-expressed TF–hydrolase networks	Identifies influential TFs linked to malting traits	Enables marker-assisted selection	(Luan, Gao, Qu, et al., 2025)

WRKY transcription factors, classically associated with stress responses, also contribute to endosperm degradation (Javed & Gao, 2023). For example, HvWRKY38 expression increases during germination and appears to regulate β-glucanase gene expression, facilitating polysaccharide breakdown (Zhou, Wang, et al., 2025). Meanwhile, bZIP TFs such as ABI5-like proteins integrate ABA and GA signaling, thereby indirectly modulating transcription of cell wall–modifying genes required for efficient reserve mobilization (Li, Luo, Wang, and Shu, 2022).

Members of the AP2/ERF family, activated during germination, further regulate cell wall hydrolase genes in response to developmental and environmental cues, reinforcing layered transcriptional control of cell wall architecture (Feng et al., 2020). Comparative transcriptomic studies between barley cultivars differing in malting performance have confirmed distinct expression patterns of these TFs, correlating with β-glucan content and malt extract yield (Hong et al., 2020, Li, Cai, Zheng, Zhang, and Ye, 2023). Bretträger et al. (2025) additionally demonstrated that the expression of glucanases and other hydrolases is tightly coordinated with physiological changes during simulated malting, emphasizing the importance of transcriptional timing in achieving optimal cell wall degradation (Bretträger et al., 2025).

Beyond transcriptional regulation, natural allelic variation at HvCslF6 and its regulatory regions provides a molecular resource for breeding barley with tailored β-glucan levels. For example, Lante et al. (2023) highlighted the functional and technological relevance of cereal β-glucans, reinforcing the potential of HvCslF6 alleles for improving malting quality. These findings underscore the value of targeting such TFs in molecular breeding. A schematic overview of the transcriptional regulation of β-glucan metabolism and cell wall remodeling, integrating GA/ABA signaling, TF families, and their downstream targets, is presented in Fig. 4. The convergence of functional genomics, transcriptomics, and co-expression network analyses suggests that manipulating key TFs through breeding or genome editing tools such as CRISPR/Cas has strong potential to optimize cell wall composition, reduce residual β-glucan, and improve brewing performance.

## 8 Transcriptional control of germination and enzyme activation

### 8.1 Crosstalk between GA and ABA pathways and transcriptional control of Cell Wall remodeling

The interplay between GA and ABA signaling pathways is fundamental to the regulation of seed germination in barley (*Hordeum vulgare*), profoundly influencing malting quality through control of genes involved in β-glucan degradation and cell wall remodeling (Hoffmannová, 2022). GA functions primarily as a promoter of germination by inducing the synthesis of hydrolytic enzymes required for mobilizing endosperm reserves, whereas ABA acts antagonistically to maintain dormancy and prevent premature germination (Zuo & Xu, 2020).

At the molecular level, GA signaling activates the transcription factor HvGAMYB, which binds to GA-responsive elements (GAREs) in the promoters of hydrolytic enzyme genes, including those encoding α-amylases and β-glucanases (Ma et al., 2024). Recent work by Li, Cai, Zheng, Zhang, and Ye (2023) using transcriptomic and chromatin immunoprecipitation sequencing (ChIP-seq) demonstrated that HvGAMYB directly regulates *HvBGLU1* and *HvEgl1*, two critical β-glucanase genes, thereby facilitating cell wall degradation during malting. Loss-of-function mutants for *HvGAMYB* exhibited reduced β-glucanase activity and delayed germination, underscoring its central regulatory role. Furthermore, HvGAMYB coordinates the expression of multiple genes involved in carbohydrate metabolism, positioning it as a master regulator of germination-related transcriptional programs (Li, Cai, Zheng, Zhang, and Ye, 2023).

ABA signaling, by contrast, is mediated through distinct transcription factors, notably ABI3 and ABI5 of the bZIP family (Han et al., 2023). HvABI5 acts as a negative regulator by repressing hydrolytic enzyme genes and antagonizing GA responses (Al-Sayaydeh et al., 2024). Collin et al. (2020) reported that HvABI5 expression remains elevated in barley grains under abiotic stress conditions that increase ABA levels, thereby prolonging dormancy and repressing germination-associated genes. They further showed that ABI5 interacts with histone deacetylases, suggesting that epigenetic repression is part of ABA-mediated dormancy control. These findings align with evidence that ABA signaling integrates environmental inputs to modulate germination via chromatin remodeling.

The balance between GA and ABA is further regulated by DELLA proteins, negative regulators of GA signaling. SLN1, the barley DELLA protein, suppresses HvGAMYB activity in the absence of GA (Sokolowska, 2021). Upon GA perception, SLN1 undergoes ubiquitin-mediated degradation, relieving repression on GAMYB and enabling activation of downstream genes (Feng, Nemzer, Devries, and Ding, 2024). Zeng et al. (2025) employed CRISPR-Cas9 to generate *sln1* mutants in barley, which exhibited precocious germination and enhanced β-glucanase expression, confirming SLN1 as a key modulator of germination timing and malt modification.

Multi-omics approaches, integrating transcriptome and metabolome data, have further clarified the crosstalk between hormonal pathways and transcriptional networks during germination. Wing Ho Ho et al. (2019) identified co-expression modules enriched in GA-responsive TFs, including MYB and NAC family members, which correlated positively with malting quality traits such as malt extract and filtration rate. Their study revealed that HvMYB61, a GA-activated MYB TF, regulates not only β-glucan biosynthesis genes such as *HvCslF6* but also cell wall remodeling enzymes, suggesting a dual role in coordinating synthesis and degradation (Wing Ho Ho et al., 2019).

In addition, AP2/ERF family TFs have emerged as integrators of hormonal and environmental cues during germination. Farooq et al. (2022) reported that specific barley ERFs are induced by GA and repressed by ABA, modulating expression of genes involved in cell wall loosening and starch degradation. Although α-amylase primarily mobilizes starch reserves, its coordinated regulation alongside cell wall–modifying enzymes ensures efficient endosperm degradation, linking carbohydrate mobilization directly with cell wall remodeling during malting. A concise summary of key transcription factors mediating GA/ABA crosstalk during germination is provided in Table 2. This table highlights their major downstream targets and functional roles in regulating β-glucan metabolism and cell wall remodeling.

**Table 2:** Key transcription factors mediating GA/ABA crosstalk during barley germination.

Regulator	Family	Key targets/processes	Role in germination	References
*HvGAMYB*	MYB	*HvBGLU1*, *HvEgl1*, α-amylase genes	Activates hydrolytic enzymes; promotes β-glucan degradation	(Garcia-Gimenez et al., 2019)
*HvABI5*	bZIP	Hydrolytic enzyme promoters; interacts with histone deacetylases	Represses germination; maintains dormancy via ABA signaling	(Zhao, He, Zhang, and Wang, 2024)
*SLN1* (*DELLA*)	GRAS	Represses *HvGAMYB*	Acts as GA–ABA balance switch; prevents precocious germination	(Meng et al., 2023), (Cheng, 2024)
HvMYB61	MYB	*HvCslF6*, cell wall hydrolases	Coordinates β-glucan synthesis and remodeling	(Shanmugaraj, 2023), (Sybilska et al., 2025)
Barley ERFs	AP2/ERF	Cell wall loosening enzymes, starch degradation genes	Integrate GA induction and ABA repression	(Hertig et al., 2023)

### 8.2 GAMYB, DOF, and bZIP: Regulation of α-amylase and hydrolase genes

The regulation of α-amylase and other hydrolase enzymes during barley malting is critically controlled by a network of transcription factors, among which GAMYB, DOF, and bZIP families play central and often coordinated roles (Li, Tan, and Zhang, 2021). These TFs integrate hormonal and developmental signals to govern the expression of genes required for starch degradation and cell wall hydrolysis, processes essential for effective malt modification.

GAMYB is a well-characterized transcription factor acting downstream of GA signaling in barley aleurone cells. GA produced in the embryo induces HvGAMYB expression, which directly activates α-amylase genes (*Amy1* and *Amy2*) by binding to GA-responsive elements (GAREs) in their promoters. Early studies established GAMYB as a master regulator (Mares et al., 2022), while more recent investigations expanded its role to additional hydrolases, including β-glucanases and proteases essential for endosperm mobilization during malting (Li, Cai, Zheng, Zhang, and Ye, 2023). The induction of GAMYB initiates a transcriptional cascade that ensures timely starch degradation and coordinated hydrolytic activity, thereby facilitating malt extract accumulation.

The DOF (DNA binding with one finger) transcription factor family provides an additional regulatory layer by modulating α-amylase expression in a GA-dependent manner (Waseem et al., 2022). DOF TFs bind to the P-box element in α-amylase promoters and interact synergistically with GAMYB to enhance transcriptional activation. For example, HvDOF17 and HvDOF19 bind to *Amy* gene promoters, and their overexpression increases α-amylase activity during germination (Zou & Sun, 2023). DOF proteins also interact with hydrolase promoters, and in some contexts, they act as repressors, fine-tuning enzyme levels depending on developmental stage or environmental conditions (Vinje & Simmons, 2024).

Members of the bZIP (basic leucine zipper) family, notably ABA-responsive factors such as ABI5, function primarily as negative regulators of hydrolase expression by mediating ABA signaling (Ghimire et al., 2024). HvABI5 binds to ABA-responsive elements (ABREs) in the promoters of α-amylase and other hydrolase genes, suppressing their GA-mediated induction during dormancy or stress conditions (Lee et al., 2022). This antagonistic regulation ensures a hormonal balance between dormancy maintenance and germination. Besides ABI5, other bZIP members integrate stress and developmental cues that affect the expression of α-amylase and cell wall–modifying enzymes (Kumar et al., 2021).

Recent transcriptomic and network analyses highlight the complexity of these interactions. Shanmugaraj (2023) demonstrated through co-expression analysis that GAMYB and DOF TFs form a regulatory module positively correlated with α-amylase expression and malt quality traits, whereas ABI5 and related bZIP TFs form an antagonistic module inversely correlated with these traits. Together, the synergistic activation by GAMYB–DOF and repression by bZIP factors dynamically modulates the timing and magnitude of α-amylase and hydrolase expression, a mechanism essential for optimizing malting performance and brewing quality.

### 8.3 TFs involved in dormancy release, embryo growth, and reserve mobilization

In malting barley, the processes of dormancy release, embryo growth, and reserve mobilization are tightly coordinated and essential for producing high-quality malt (Rani & Bhardwaj, 2021). These developmental transitions are governed by a complex transcriptional network that integrates hormonal signaling and environmental cues, ensuring efficient germination and seedling establishment. Dormancy release is primarily regulated by the balance between ABA and GA (Wang and Han, 2025). Transcription factors from the bZIP family, particularly ABI3 and ABI5, act as repressors of germination by maintaining ABA-mediated dormancy (Wu et al., 2020). In barley, reduction of HvABI5 activity is critical for dormancy release and initiation of germination. Moreover, HvABI5 interacts with histone deacetylases and chromatin remodelers, linking ABA signaling to epigenetic repression of germination-related genes (Li, Li, Wang, Zhang, and Wang, 2022, Liu et al., 2022). As ABA signaling declines, rising GA levels initiate embryo growth.

During embryo growth, GA signaling activates the transcription factor *HvGAMYB*, a major positive regulator that induces the expression of genes encoding α-amylases, proteases, and β-glucanases. These enzymes mobilize starch and cell wall reserves in the endosperm, releasing nutrients for the expanding embryo (Li, Cai, Zheng, Zhang, and Ye, 2023, Ogrodowicz et al., 2023). GAMYB activity is suppressed by DELLA proteins such as SLN1, which restrain GA responses until conditions favor germination, ensuring precise developmental timing. Members of the NAC family, including *HvNAC005*, also support embryo expansion by promoting the expression of expansins and cell wall–modifying enzymes, reinforcing GA-mediated tissue remodeling (Fuertes-Aguilar & Matilla, 2024).

Reserve mobilization is fine-tuned by multiple TF families. DOF transcription factors cooperate with GAMYB to regulate α-amylase gene expression, adjusting enzyme production to meet metabolic demand during germination (Fuertes-Aguilar & Matilla, 2024, Lee, Yoon, and Hwang, 2022). WRKY TFs, such as HvWRKY38, regulate β-glucanase and stress-response genes, thereby coordinating endosperm degradation with seedling vigor under variable environments (Li, Khoso, et al., 2024). Similarly, members of the AP2/ERF family function as integrators of hormonal and stress signals, modulating the expression of cell wall hydrolases and starch-degrading enzymes to ensure efficient reserve mobilization and improved malt quality (Ding et al., 2025). Collectively, transcription factors from the bZIP (ABI3, ABI5), MYB (GAMYB), NAC, DOF, WRKY, and AP2/ERF families coordinate dormancy release, embryo growth, and reserve mobilization in barley. Dissecting this regulatory framework offers promising strategies to enhance malting efficiency and brewing quality through targeted breeding and functional genomics.

## 9 Future perspectives

Looking to the future, several promising avenues could improve our understanding of transcription factor regulation in malting barley and facilitate translation into improved malt quality. One important direction is the development of dynamic models and network simulations to capture how transcription factors interact during germination and mobilization of reserves over time. Such computational approaches can unravel complex feedback loops and signaling cascades, and reveal critical regulatory junctions and timing mechanisms. By integrating multi-level experimental datasets, these models could guide targeted experiments and ultimately enable precise control of transcriptional programs associated with germination (Pazhamala et al., 2021).

Machine learning and explainable AI are also powerful tools to analyze large transcriptome datasets and identify the most influential transcription factors and gene networks associated with malt quality. By analyzing gene expression profiles in different barley genotypes and in different environments, these algorithms can predict candidate regulators and prioritize them for functional validation. Advances in explainable AI will improve the interpretability of these predictions, and clarify why certain genes are emphasized and how they can be manipulated in breeding or biotechnology (Danilevicz et al., 2025, Feng, Gao, and Wang, 2024).

Another priority is transcriptome analysis in different environments. As malting barley is grown and processed under a range of climatic and industrial conditions, it is important to determine which transcriptional networks remain stable and which adapt to stress factors such as temperature fluctuations, water availability, or pathogen pressure. Identifying transcription factors that consistently support favorable malting performance in different environments will be critical for breeding resilient, climate-adapted varieties that maintain high quality under variable conditions (Mangaraj et al., 2025).

Synthetic biology offers a new platform for precisely adjusting the activity of transcription factors. By using CRISPR/Cas genome editing and synthetic promoters, it becomes possible to control the spatial and temporal activity of regulators such as *HvGAMYB*. This could enable optimized enzyme production and efficient mobilization of reserves tailored to specific malting requirements, improving the consistency and efficiency of malt production (Abebaw & Tobiaw, 2025, Huang, Hilscher, Stoger, Christou, and Zhu, 2021). Together, these approaches of dynamic modeling, machine learning, multi-environment transcriptomics, and synthetic biology provide an integrated roadmap for future advances. By combining computational prediction with functional validation, researchers and breeders can develop next-generation barley varieties with improved malt quality, resilience, and adaptability to meet the demands of a changing climate and brewing industry.

## 10 Conclusion

Transcription factors act as central regulators of barley malting, and coordinate processes ranging from the initiation of dormancy and embryonic development to the mobilization of reserves and the remodeling of cell walls. Families such as MYB (e.g., GAMYB), NAC, DOF, bZIP, WRKY, and AP2/ERF control the expression of genes encoding hydrolases, modifiers of storage proteins and cell wall degrading enzymes, thus ensuring timely and efficient modification of the endosperm. Systems biology approaches that integrate genomics, transcriptomics, and computational modeling are beginning to reveal how these transcription factors interact with hormonal signaling pathways such as gibberellin and ABA, as well as with environmental factors during germination and malting. Such multi-layered analyses are crucial for identifying the key regulatory nodes that determine malt quality traits, including enzyme activity, starch degradation, and β-glucan degradation. Translating these insights into molecular breeding and genome editing strategies provides a pathway to develop barley varieties with improved malting performance, enhanced resilience to environmental stress, and higher consistency of brewing quality. A deeper mechanistic understanding of transcription factor networks will therefore be central to both basic research and the practical improvement of barley for the malting and brewing industries.

## CRediT authorship contribution statement

**Bahman Panahi:** Writing – review & editing, Writing – original draft, Data curation, Conceptualization. **Rasmieh Hamid:** Writing – review & editing, Writing – original draft. **Zahra Ghorbanzadeh:** Writing – original draft, Validation. **Saber Golkari:** Writing – review & editing, Writing – original draft. **Mehmet Yildirim:** Writing – review & editing, Writing – original draft. **Feba Jacob:** Writing – review & editing.

## Ethics approval and consent to participate

Not applicable. This article is a review and does not involve new experiments requiring ethics approval or consent to participate.

## Funding

This study was funded by Agricultural Biotechnology Research Institute of Iran (ABRII) with grant numbers 0138-05-0503-029-0003-03022-030545.

## Declaration of competing interest

The authors declare that they have no known competing financial interests or personal relationships that could have appeared to influence the work reported in this paper.

## Data Availability

Data will be made available on request.
